# Copenhagen study of overweight patients with coronary artery disease undergoing low energy diet or interval training: the randomized CUT-IT trial protocol

**DOI:** 10.1186/1471-2261-13-106

**Published:** 2013-11-19

**Authors:** Lene Rørholm Pedersen, Rasmus Huan Olsen, Marianne Frederiksen, Arne Astrup, Elizaveta Chabanova, Philip Hasbak, Jens Juul Holst, Andreas Kjær, John W Newman, Rosemary Walzem, Ulrik Wisløff, Ahmad Sajadieh, Steen Bendix Haugaard, Eva Prescott

**Affiliations:** 1 Department of Cardiology, Bispebjerg University Hospital, Copenhagen, Denmark; 2 Department of Nutrition, Exercise, and Sports, Faculty of Science, University of Copenhagen, Copenhagen, Denmark; 3 Department of Diagnostic Radiology, University Hospital of Herlev, Copenhagen, Denmark; 4 Rigshospitalet, Department of Clinical Physiology, Nuclear Medicine & PET, Copenhagen, Denmark; 5 Faculty of Health and Medical Sciences, Department of Biomedical Sciences, Endocrinology Research Section, University of Copenhagen, Copenhagen, Denmark; 6 Obesity and Metabolism Research Unit, USDA-ARS-Western Human Nutrition Research Center, Davis CA, USA; 7 Faculty of Nutrition, Texas A&M University, College Station, TX, USA; 8 K.G. Jebsen Center of Exercise in Medicine at Department of Circulation and Medical Imaging, Norwegian University of Science and Technology, Trondheim, Norway; 9 Department of Internal Medicine, Amager University Hospital, Copenhagen, Denmark; 10 Clinical Research Centre, Hvidovre University Hospital, Copenhagen, Denmark

**Keywords:** Coronary artery disease, Secondary prevention, Rehabilitation, Exercise, Overweight, Obesity, Weight loss, Life style changes

## Abstract

**Background:**

Coronary artery disease (CAD) is accountable for more than 7 million deaths each year according to the World Health Organization (WHO). In a European population 80% of patients diagnosed with CAD are overweight and 31% are obese. Physical inactivity and overweight are major risk factors in CAD, thus central strategies in secondary prevention are increased physical activity and weight loss.

**Methods/Design:**

In a randomized controlled trial 70 participants with stable CAD, age 45–75, body mass index 28–40 kg/m^2^ and no diabetes are randomized (1:1) to 12 weeks of intensive exercise or weight loss both succeeded by a 40-week follow-up. The exercise protocol consist of supervised aerobic interval training (AIT) at 85-90% of VO_2_peak 3 times weekly for 12 weeks followed by supervised AIT twice weekly for 40 weeks. In the weight loss arm dieticians instruct the participants in a low energy diet (800–1000 kcal/day) for 12 weeks, followed by 40 weeks of weight maintenance combined with supervised AIT twice weekly. The primary endpoint of the study is change in coronary flow reserve after the first 12 weeks’ intervention. Secondary endpoints include cardiovascular, metabolic, inflammatory and anthropometric measures.

**Discussion:**

The study will compare the short and long-term effects of a protocol consisting of AIT alone or a rapid weight loss followed by AIT. Additionally, it will provide new insight in mechanisms behind the benefits of exercise and weight loss. We wish to contribute to the creation of effective secondary prevention and sustainable rehabilitation strategies in the large population of overweight and obese patients diagnosed with CAD.

**Trial registration:**

ClinicalTrials.gov: NCT01724567

## Background

Coronary artery disease (CAD) is accountable for more than 7 million deaths each year according to the World Health Organization (WHO) [1]. Overweight and physical inactivity are major risk factors in CAD. Weight loss through reduced calorie intake and improved fitness through increased physical activity are central strategies in both primary and secondary prevention. CAD mortality has decreased considerably during the last 30 years due to improved treatment, prevention and rehabilitation [2]. However, due to the increased life expectancy the prevalence of CAD is increasing; thus, effective secondary prevention, optimal patient advice and sustainable rehabilitation programs are essential in decreasing morbidity and increasing quality of life in CAD.

### Benefits of exercise training on cardiovascular risk factors

Peak aerobic capacity (VO_2_peak) is a predictor of all-cause mortality in patients with CAD [3]. Several smaller studies in patients with metabolic syndrome [4], CAD [5], congestive heart failure (CHF) [6], hypertension [7] and diabetes [8] have shown that aerobic interval training (AIT) is superior to moderate continuous training in improving VO_2_peak and other cardiovascular risk markers such as endothelial function, glucose tolerance, hypertension and dyslipidemia. Furthermore, longer-term exercise training may even be superior to percutaneous coronary intervention (PCI) with stent implantation on event-free survival, anti-inflammatory effect and cost-effectiveness in selected patients with stable CAD [9].

### Benefits of weight loss on cardiovascular risk factors

Obesity is a modifiable, independent risk factor of cardiovascular disease [10]. In a European population 80% of the patients diagnosed with CAD are overweight (Body Mass Index (BMI) > 25 kg/m^2^) and 31% are obese (BMI > 30 kg/m^2^) [11]. In overweight but otherwise healthy subjects, a low energy diet (LED), defined as 800 – 1000 kcal/day, resulted in a considerable weight loss with no apparent risk [12]; however, there are no studies describing the use of LED in patients with CAD. The pan-European study DiOGenes used an LED to facilitate an initial weight loss of more than 8% of the original body weight in an overweight population, which resulted in a reduction of both cardiovascular [13] and metabolic risk [14]. A British study using a very low energy diet (600 kcal) to induce weight loss showed that insulin resistance and beta cell failure could be reversed in diabetic patients [15]. Reduced heart rate variability (HRV) is an established marker of cardiovascular risk. In the COBRA study [16], a 5% weight loss in postmenopausal obese women led to an increase in HRV.

A considerable challenge is to maintain a stable weight following the initial weight loss. According to the DiOGenes study, the most effective way to achieve this is ad libitum consumption of a diet with high protein content and low glycemic index carbohydrates [17]. The effect of this diet prescription in CAD patients is not well described; however, the participants in the DiOGenes study obtained reductions in triglycerides and high sensitivity C-reactive protein (hsCRP) while HDL cholesterol was significantly increased after 26 weeks’ diet [13].

### The effect of lifestyle changes

Large interventional studies aiming at weight reduction and improved fitness in overweight subjects have found a favorable effect on cardiometabolic risk following intervention [18,19]. The recent look AHEAD trial showed improved fitness and glycemic control and a reduction in weight and cardiovascular risk factors in overweight patients with type II diabetes undergoing lifestyle intervention compared with diabetes support and education after one [20] and four [21] years’ intervention. Nonetheless, improvements were difficult to sustain over a prolonged period and there was no difference in cardiovascular morbidity and mortality after a median follow-up of 9.6 years [22]. A Danish study from 2012 comparing endurance training with or without weight loss and weight loss obtained by calorie restriction in sedentary, moderately overweight but otherwise healthy young men found that loss of fat mass improved hepatic insulin sensitivity, while peripheral insulin sensitivity and VO_2_peak were improved only by endurance training [23].

### Coronary flow reserve, weight loss and exercise

Coronary flow reserve (CFR), which is a measure of coronary microvascular function in the absence of significant stenosis, is reduced in individuals with diabetes [24], obesity [25] and CAD [26]. CFR has been shown to be a predictor of poor prognosis and mortality in patients with known or suspected CAD even when the coronary arteries were normal [27,28]. In CHF patients CFR has been shown to be positively correlated to VO_2_peak [29]. Exercise training has been shown to improve CFR in patients with CAD [30], while weight reduction following a multidisciplinary rehabilitation program resulted in improved CFR in overweight persons with no CAD [25]. To our knowledge, there are no interventional studies in CAD patients comparing the effect of weight loss and exercise on CFR.

### Weight loss, exercise and insulin resistance

Insulin resistance is directly associated to CAD [31]. Physical exercise and weight loss have been shown to improve insulin action [19], which is considered an important mechanism behind the reduction in cardiovascular risk in CAD with lifestyle modification. Presence of abdominal adipose tissue particularly is associated with insulin resistance and thus cardiovascular risk via pathways involving adipokines, cytokines, ceramides and dysfunctional fatty acid composition [32].

Incretin hormones, released from the small intestine, play an important role in insulin secretion and resistance. Incretin secretion is impaired in patients with diabetes and smaller reductions in secretion are seen in overweight and obese individuals with insulin resistance and impaired glucose tolerance [33]. Incretin hormones facilitate the glucose uptake in the myocardium and are possibly associated with left ventricular function in CAD [34]. This could imply that changes in incretin function in overweight individuals following weight loss play a role in changes in cardiac and endothelial function. Small studies have indicated that physical exercise could influence incretin secretion [35], however, knowledge is limited regarding the effect of exercise and weight loss on incretin function as well as the metabolic and hormonal effect in adipose tissue.

### Rationale of the study

Several benefits from weight loss and exercise training on cardiovascular and diabetes risk factors have been described. Both LED and AIT could be first choice in secondary prevention in patients with CAD, yet a head-to-head comparison has not yet been performed. Thus, the primary objective of the CUT IT study is to investigate the effect of an effective weight loss protocol compared to 12 weeks of intensive AIT in overweight CAD patients. The primary endpoint is change in CFR and secondary endpoints describe the effect of the intervention on cardiovascular, metabolic and inflammatory risk markers. Secondary objectives are sustainability of the results during a 40-week follow-up period, containing AIT twice weekly or weight maintenance and AIT twice weekly.

## Methods/Design

### Study population

**Figure 1:**
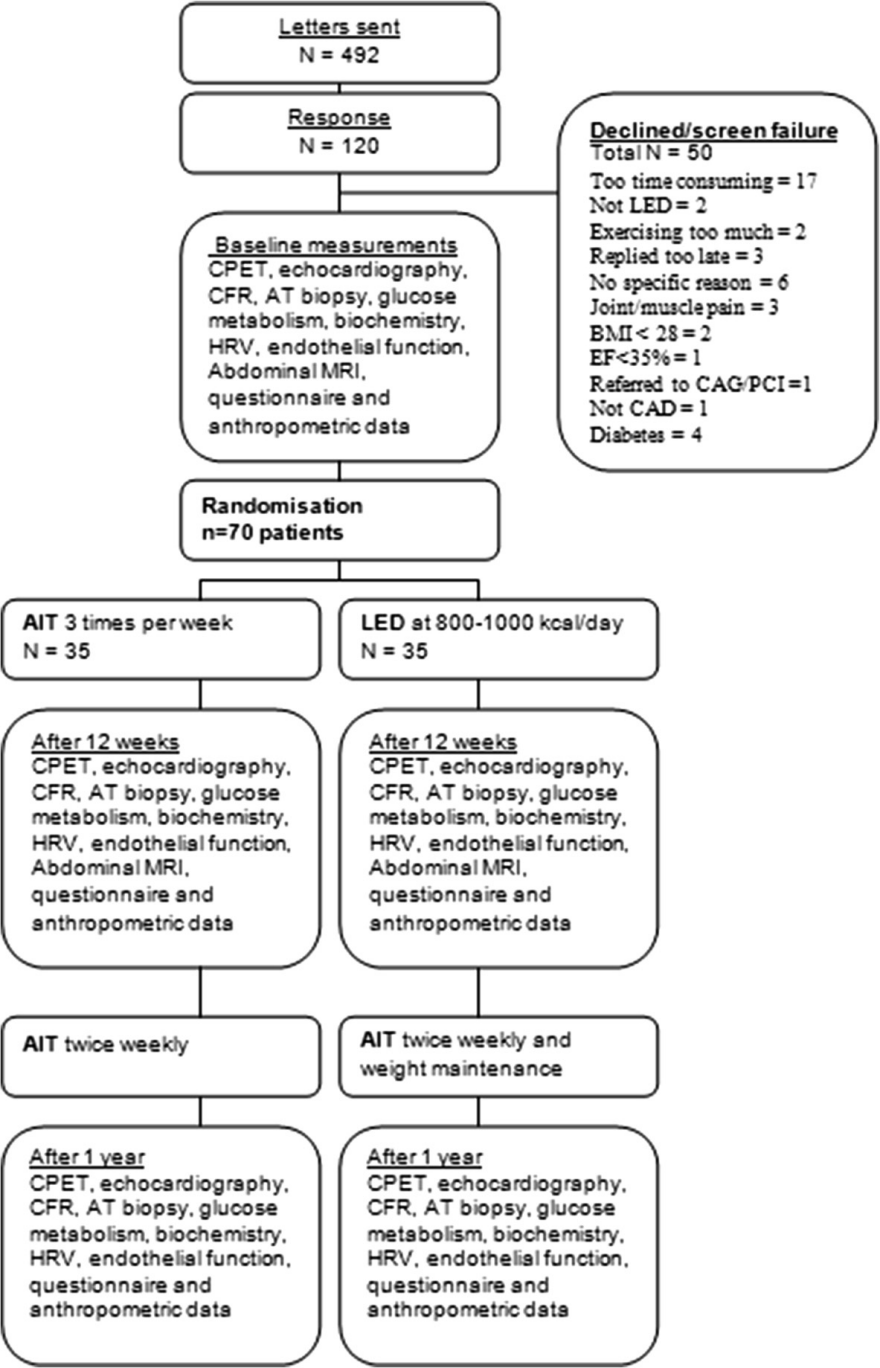
Study design and inclusion.

Participants were included in the study from September 2011 to April 2013. The study is on-going and the last participants will be evaluated in April 2014. We included 70 patients (Figure 1) with stable CAD; age 45–75 years, BMI 28–40 kg/m^2^ not diagnosed with diabetes. Further inclusion and exclusion criteria are listed in Table 1. The participants were found through the coronary rehabilitation unit at Bispebjerg University Hospital and a registry of coronary angiography, which comprises all angiographies performed in the Danish Capital Region. If the medical record did not reveal any exclusion criteria, a letter of invitation was sent with written information about the trial. People interested in participation were invited to a screening visit, conducted by a medical doctor connected to the study, where they received information about the study orally. To screen for certain exclusion criteria echocardiography and cardiopulmonary exercise test (CPET) including ECG were performed. Furthermore, blood samples were taken to screen for diabetes, kidney- and liver disease. If eligible and still interested the participant signed informed consent.

**Table 1:** Inclusion and exclusion criteria

Inclusion criteria	Stable CAD (defined as previous MI, PCI, CABG > 6 months prior to inclusion or classic angina pectoris and positive angiogram/SPECT/CT-angiogram)
	Age 45–75 years
	BMI 28–40 kg/m^2^
Exclusion criteria	Known diabetes
	Diabetes diagnosed at screening (repeated fasting plasma glucose ≥ 7 mM or Hba1c > 7%)
	Severe or moderate valve disease
	Main stem stenosis
	Ejection Fraction < 35%
	Physical or mental disability - expected to prevent completion of intervention
	Severe COPD or asthma (FEV1 < 50% of expected)
	Active cancer
	Severe kidney (eGFR < 40 mL/hour) or severe liver disease
	Severe ischemia or arrhythmias during exercise test
	2. or 3. degree AV block, not protected by pacemaker
	Organised training more than 2 times a week prior to inclusion
	Significant weight loss or weight gain (> 5%) 3 month prior to inclusion

*Abbreviations*: *BMI* Body Mass Index, *CAD* Coronary Artery Disease, *COPD* Chronic Obstructive Pulmonary Disease, *FEV1* Forced Expiratory Volume the 1st second, *eGFR* estimated glomerular Filtration Rate, *MI* Myocardial Infarction, *PCI* Percutaneous Coronary Intervention, *CABG* Coronary Artery Bypass Graft.

### Randomization and stratification

The participants were consecutively enrolled during the inclusion period and randomized (1:1) into two groups:

(1) 12 weeks of AIT three times a week, followed by 40 weeks’ AIT twice weekly.

(2) 8–10 weeks’ LED followed by 2–4 weeks of transition to a high protein/low glycemic index diet and 40 weeks of weight loss maintenance and AIT twice weekly.

Randomization was stratified according to BMI (≤ 32.5; > 32.5). A third party unrelated to the study performed en bloc randomization with bloc size 2, 4 and 6 using Stata 11.1 software (StataCorp, 4905 Lakeway Drive, College Station, TX, USA).

### Intervention

#### Aerobic interval training

The patients warm up at a moderate intensity for 10 minutes on a staircase (total of 73 steps, minimum 5 times up and down) or on an exercise bike, followed by high intensity interval training (85–90% of VO_2_peak as determined by CPET, Borg scale 17–18) on an exercise bike. The intervals are 1–4 minutes, with a total of 16 minutes, separated by active pauses of 1–3 minutes. The total duration of each training session is 38 minutes. Physiotherapists with experience in cardiac rehabilitation instruct the participants and supervise all training session. Training intensity is monitored using heart rate monitors, lactate measurements and the Borg Scale.

#### Low energy diet

The participants are instructed by experienced clinical dieticians to maintain an LED for 8–10 weeks. The LED consisted of soups, shakes, porridge and bars from the Cambridge Weight Plan (Northants, UK) 4 times a day, combined with a few additional vegetables, yoghurt and one litre of skimmed milk resulting in a total of 800–1000 kcal/day. The diet is designed to provide the participants with all necessary micro- and macronutrients and has previously been shown to be efficient in weight loss [36]. The consultations with the dieticians are both individual and group sessions at study week 1, 2, 4, 6, 8 and 10. At study week 8 or 10 the participants are introduced to the maintenance diet.

#### Weight maintenance diet

The weight maintenance diet is a high protein/low glycemic index diet modified from the DiOGenes study [17]. Taking into consideration that the participants have CAD the high protein diet was adapted to resemble the Mediterranean diet, which is recommended to heart patients [2], thus the protein sources were mainly fish, poultry, egg, dairy, and vegetables. The transition to the maintenance diet is conducted during visits at week 8, 10 and 12 and hereafter the participants consult the dietician monthly.

### Baseline and follow-up examinations

All participants are examined at baseline, after 12 weeks and after a year (flowchart summarized in Figure 1). Most examinations were performed at University Hospital of Bispebjerg, Department of Cardiology, except the MRI that was performed at University Hospital of Herlev and PET that was performed at Rigshospitalet. The primary endpoint is change in CFR following the first 12-weeks’ intervention. Coronary flow velocity (CFV) is measured using transthoracic Doppler echocardiography to determine the flow in the distal part of the left anterior descending (LAD) coronary artery. CFR is calculated as the ratio between CFV at rest and during hyperaemia induced by infusion of dipyridamole or adenosine. Cardiac positron emission tomography-computed tomography (PET/CT) is used as the “gold standard” to validate CFR in a sample of the population.

Secondary endpoints will provide cardiovascular and metabolic insight into the effects of exercise and weight loss:

#### Cardiovascular endpoints

• Aerobic exercise capacity assessed by VO_2_peak

• Systolic and diastolic function assessed by echocardiography at rest and during semi-supine exercise

• Heart rate variability and ischaemic burden assessed by 48 hours Holter monitoring

• Peripheral endothelial function assessed digitally using the Endopat2000 [37]

• Blood pressure

#### Metabolic and inflammatory endpoints

• Beta cell function, insulin production and response expressed as Matsuda-index [8] and HOMA-index and assessed using a 3-hour oral glucose tolerance test (OGTT)

• Incretin hormone response measured during a 3-hour OGTT

• Non-esterified fatty acids response measured during the 3-hour OGTT

• Inflammatory markers: interleukin-6 (IL-6), tumour necrosis factor alpha (TNF-α), high sensitive C-reactive protein (hsCRP) and soluble urokinase plasminogen activating receptor (suPAR)

• Fasting triglycerides and lipoproteins

• Oxidized lipids, lipoprotein particle size and density distribution

• Adipokines

• Body composition assessed by anthropometry and dual X-ray absorptiometry (DEXA)

• Visceral, liver and ectopic muscle fat assessed by abdominal magnetic resonance imaging (MRI)

• Fatty acids composition, ceramides and cytokine mRNA expression in adipose tissue

#### Other parameters

• Physical activity assessed by accelerometer and International Physical Activity Questionnaire (IPAQ)

• Changes in diet assessed by a 3-day written diet record

• Anxiety and depression symptoms assessed by the Hospital Anxiety and Depression (HADS) questionnaire

### Statistics

The primary endpoint was CFR and we wished to be able to detect difference in change in CFR between groups of 10%, equal to a change in CFR of 0.24 assuming a baseline mean CFR of 2.4 in similar patient groups from our experience [25,26,38]. Information on the expected standard deviation (SD) of the within individual change was not available and was therefore conservatively assumed to be 0.3. With power of 0.8 and two-sided significance level of 0.05, 26 patients should be included in each group. Anticipating 30% dropout, randomization of a total of 70 patients was chosen. Based on previous studies, this sample size is sufficient to detect relevant differences in endothelial function [39,40], HRV [41], Matsuda index [8] and HOMA [42]. With a SD of CFR of 0.6, this sample size will provide 80% power to detect a between group difference of 0.5 in CFR after the intervention.

Analyses will be by intention-to-treat whenever possible and care will be taken to encourage drop-outs to participate in follow-up examinations.

### Ethics and dissemination

The study complies with the Declaration of Helsinki and has been approved by the Regional Ethics Committee of the Capital Region in Denmark (no H-4-2012-146) and the Danish Data Protection Agency (no 2011-41-6313). Participants gave oral and written informed consent and are free to decline or retract participation at any time. All data are handled confidentially and the participants are ensured anonymity. The trial is registered at clinicaltrials.gov (NCT01724567). Our findings will be published in peer-reviewed journals and presented at national and international congresses.

Most of the procedures are used in everyday clinical practice and the side effects are temporary. The participants will experience some discomfort related to the adipose tissue biopsy and during infusion with dipyridamole/adenosine used to induce stress during the CFR assessment (echocardiography and PET/CT). The study will provide knowledge on the optimal composition of effective rehabilitation programs to help the large group of overweight patients with CAD. Furthermore, we expect each participant to benefit directly from the intervention independent of which study arm they are randomized to.

## Discussion

The CUT IT trial is a single-center, randomized, interventional trial with consecutive inclusion of participants. In- and exclusion criteria have been selected trying to balance the wish to have high external validity, yet without creating a population with too much heterogeneity. Randomization will limit selection bias. Monitoring with accelerometers, IPAQ and diet records are performed in order to assess if participants in the weight loss arm change their physical activity level and if the participants in the exercise arm change their diet during the intervention period. Concerning external validity we must also consider that people agreeing to participate in an intervention study, are more motivated to lifestyle changes than a general population of patients with CAD. Analysis of non-participants from registry data will assess the size of potential bias.

The primary endpoint is CFR assessed by transthoracic Doppler echocardiography. This non-invasive measure of CFR has been validated against invasive measurements [43,44] and PET [45]. CFR is performed at our research unit with a low intra- and inter-observer variability and good repeatability [29,46].

Blinding of participants and investigators is an important issue in clinical trials. In this study blinding of the participants to the intervention is not possible. Blinding of the investigator in examinations involving contact to the participants is difficult, since it is possible to distinguish the participants in the weight loss group from the ones in the exercise group, due to the expected weight loss. This is relevant concerning echocardiography and exercise testing. The same echo-cardiographer will perform all the examinations to prevent inter-observer variation and a person blinded to the intervention will perform the off-line echo analyses. In exercise testing we try to limit bias by using standardised written instructions for each test and to blind the investigator to the result of baseline test.

Biochemical analysis of adipose tissue and plasma samples and Holter analysis will be blinded to randomization. Performance and analysis of MRI and PET/CT-scan will be blinded to randomization. Endothelial function is calculated by the Endopat2000 software independently of the person performing the examination.

## Conclusion

We aim to undertake a head-to-head comparison of AIT along with rapid weight loss followed by AIT on cardiovascular risk markers and cardiac function in overweight patients with CAD. The study will, furthermore, provide important mechanistic insights of both short- and long-term effects of the two interventions. Importantly, the study will provide guidance on best rehabilitation strategies in the large number of overweight and obese patients suffering from CAD.

## Competing interests

The Cambridge Weight Plan supplied the LED, but had no influence on the design of the study and will not have any influence on the analysis and interpretation of the results.

## Authors’ contributions

LRP, RHO, EP and SH designed the study and drafted the manuscript. LRP and RHO are daily coordinators of the trial with supervision from EP and SH. The remaining co-authors contributed to the study design on the following subjects: MF and UW: exercise intervention; AA: diet intervention; EC: abdominal MRI; PH and AK; PET/CT; JJH, incretins; JN and RW, plasma lipoproteins and adipose tissue biopsies, AS: Holter monitoring. The manuscript was read, revised and finally approved by all co-authors.

## Pre-publication history

The pre-publication history for this paper can be accessed here:

http://www.biomedcentral.com/1471-2261/13/106/prepub
